# Quantitative measures of vortex veins in the posterior pole in eyes with pachychoroid spectrum diseases

**DOI:** 10.1038/s41598-020-75789-w

**Published:** 2020-11-11

**Authors:** Hidetaka Matsumoto, Junki Hoshino, Yosuke Arai, Ryo Mukai, Kosuke Nakamura, Yuka Kikuchi, Shoji Kishi, Hideo Akiyama

**Affiliations:** Department of Ophthalmology, School of Medicine, Gunma University, 3-39-15 Showa-machi, Maebashi, Gunma 371-8511 Japan

**Keywords:** Diseases, Medical research, Neurology

## Abstract

Pachychoroid spectrum diseases have attracted increasing attention, though their pathophysiology has yet to be fully elucidated. In this study, we assessed the vascular diameters of vortex veins in pachychoroid spectrum diseases such as central serous chorioretinopathy (CSC), pachychoroid neovasculopathy without polypoidal lesions (PNV), and pachychoroid neovasculopathy with polypoidal lesions (polypoidal choroidal vasculopathy: PCV). In a retrospective case series of 94 eyes with CSC, 60 eyes with PNV and 57 with PCV, we binarized en face optical coherence tomography (OCT) images of choroidal vortex veins and analyzed the mean diameter of vortex veins. The presence of anastomosis between the superior and inferior vortex veins and central choroidal thickness (CCT) were also evaluated using OCT images. CSC showed significantly larger mean diameter of vortex veins than PCV (P < 0.05). Anastomosis between superior and inferior vortex veins was observed in over 90% of eyes with each pachychoroid spectrum disease. The patients with CSC were the youngest, followed by PNV patients, and then patients with PCV. The largest CCT values were observed in CSC eyes, followed by PNV eyes, and then PCV eyes. CCT correlated with the mean diameter of vortex veins (rs = 0.51, P < 0.01). These findings suggest that congestion of vortex veins might show gradual amelioration corresponding to the development of anastomosis between the superior and inferior vortex veins during the course of progression of pachychoroid spectrum diseases. Moreover, the mean diameter of vortex veins can be used as a parameter indicating choroidal congestion.

## Introduction

The term “pachychoroid” means choroidal thickening associated with dilatation of outer choroidal vessels, so-called pachyvessels^1,2^. Recently, pachychoroid has attracted increasing attention, in both the clinical and basic research fields of ophthalmology^3^. To date, several pachychoroid spectrum diseases including central serous chorioretinopathy (CSC), pachychoroid neovasculopathy (PNV), and polypoidal choroidal vasculopathy (PCV) have been reported^4–6^. It has been speculated that CSC might progress to PNV, and then ultimately to PCV^5,7^. Moreover, pachychoroid is thought to have major involvement in the development of age-related macular degeneration (AMD) in Asian populations^8^.

Optical coherence tomography (OCT) is a powerful tool for noninvasive evaluation of diseases affecting the ocular fundus, including the pachychoroid spectrum diseases. Imamura et al. first reported the choroidal thickening in eyes with CSC utilizing the enhanced depth imaging of B-mode OCT^4^. They suggested that increased hydrostatic pressure in the choroid might be a cause of CSC^4^. Sonoda et al. reported a binarization method for B-mode OCT images of the choroid which allowed the luminal and stromal areas to be distinguished^9^. Using this method, they found dilatation of outer choroidal vessels to contribute to the choroidal thickening characteristic of CSC^9^. Moreover, the same group applied the binarization method to en face OCT images of the choroid to investigate the features of choroidal vessels in Haller’s layer^10^. They analyzed several parameters including vessel area, vessel length, and mean vessel diameter employing en face OCT images depicting Haller’s layer^10^.

Several reports have suggested vortex vein congestion to be a cause of pachychoroid^11,12^. Using en face OCT images of the choroid, Hiroe and Kishi reported an asymmetric pattern of superior and inferior vortex veins in CSC^13^. In a previous study, we demonstrated that the area of pachyvessels overlapped with the area of filling delay on indocyanine green angiography (ICGA) in CSC^14^. We also reported that anastomosis between superior and inferior vortex veins was more frequently observed in eyes with pachychoroid spectrum diseases, including CSC, PNV and PCV, than in normal eyes^15,16^. Moreover, choroidal neovascularization (CNV) was reportedly present above the pachyvessels in PNV and PCV^5,6,15^. These reports support the assumption that congestion and remodeling of choroidal vessels might be related to the development of pachychoroid spectrum diseases. However, other studies found that excessive stimulation of the sympathetic nerve might be a cause of CSC^17,18^. Therefore, further studies are needed to elucidate the pathophysiology of pachychoroid development and, thereby, to establish optimal treatments.

Herein, using a binarization method, we quantitatively analyzed en face OCT images of choroidal vortex veins in eyes with CSC, PNV, and PCV.

## Results

**Figure 1:**
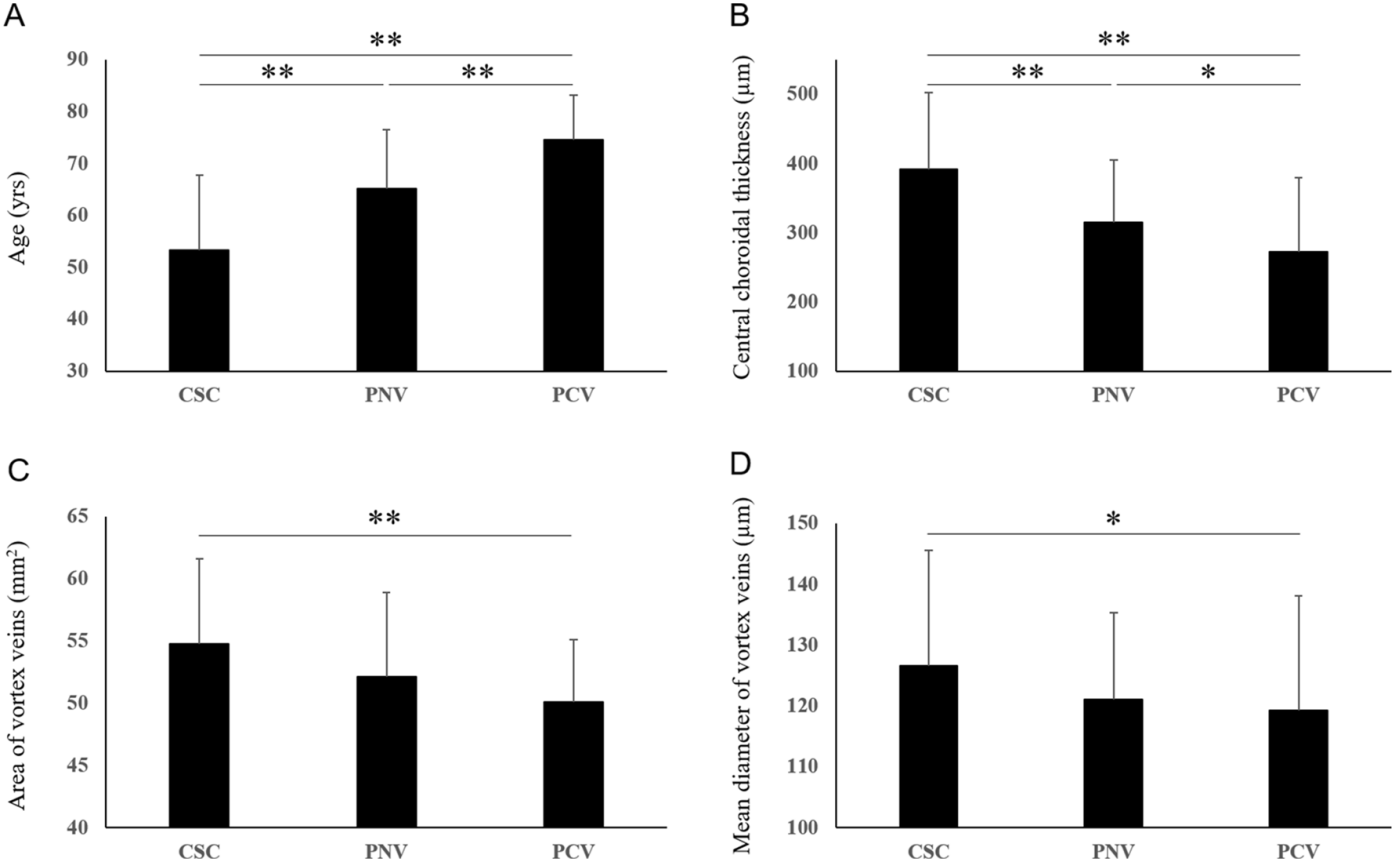
Comparison of age, central choroidal thickness, area of vortex veins, and mean diameter of vortex veins in patients with pachychoroid spectrum diseases. Data are presented as average ± standard deviation (*P < 0.05, **P < 0.01).

**Figure 2:**
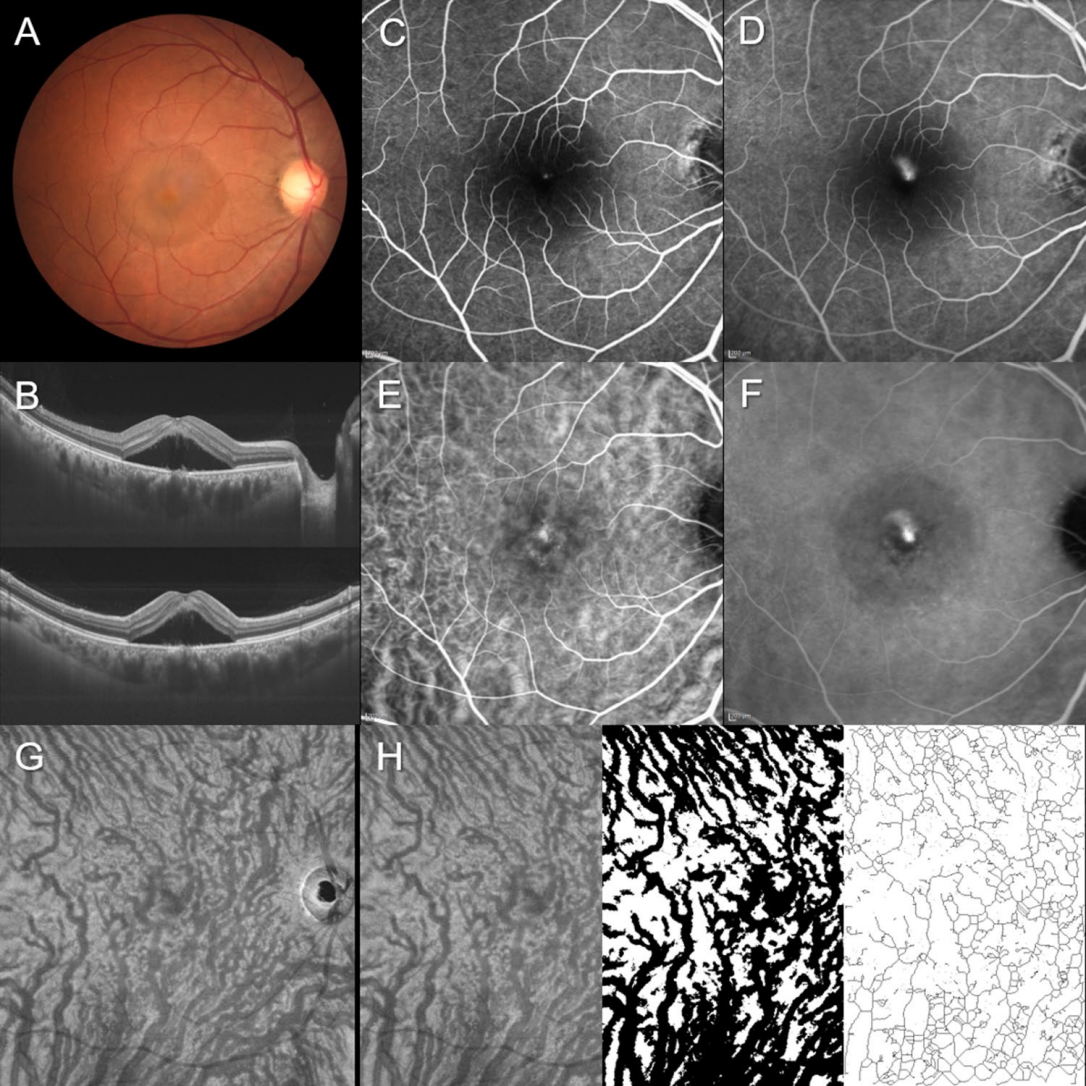
Images of the right eye of a 40-year-old man with central serous chorioretinopathy. The refraction was 0.00 diopters. Best-corrected visual acuity was 0.40 logarithm of the minimum angle of resolution unit. (**A**) Color fundus photograph shows a serous retinal detachment (SRD) at the macular area. (**B**) 12 mm horizontal and vertical B-mode optical coherence tomography (OCT) images through the fovea show pachychoroid with dilated outer choroidal vessels (vortex veins) associated with SRD. The central choroidal thickness is 493 µm. (**C**,**D**) Fluorescein angiography (early and late phases) shows smoke-stack pattern dye leakage at the fovea. (**E**,**F**) Indocyanine green angiography (early and late phases) shows dilated choroidal vessels and dye leakage at the fovea. (**G**) En face OCT image (12 mm × 12 mm) showing dilated vortex veins in the deep layer of the choroid. Horizontal watershed is lost because of the anastomoses between the superior and inferior vortex veins. (**H**) En face OCT image (temporal 8 mm × 12 mm), binarized image, and skeletonized image. The area, length, and mean diameter of vortex veins are 56.6  mm^2^, 415 mm, and 136 µm, respectively.

**Figure 3:**
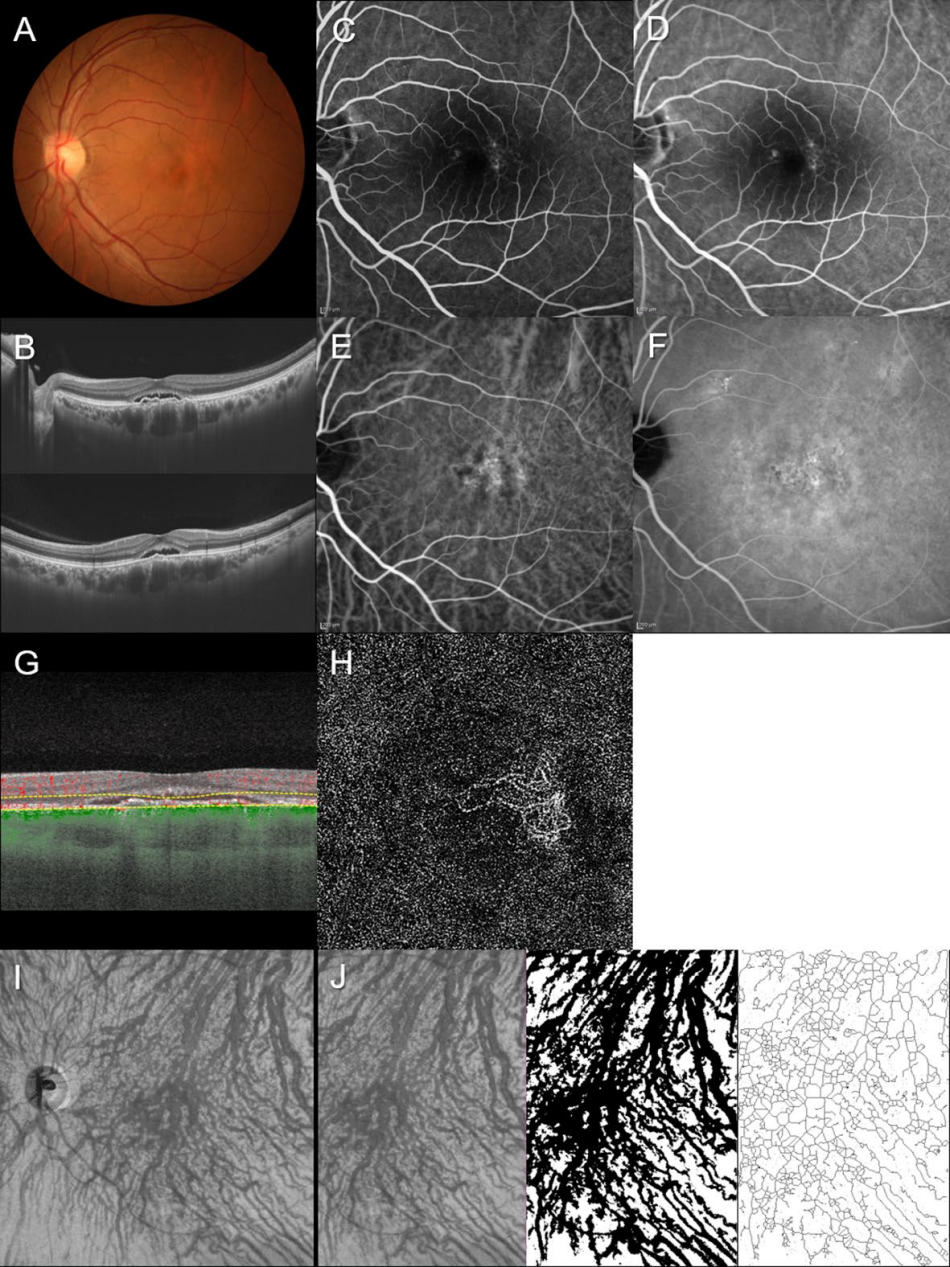
Images of the left eye of a 55-year-old man with pachychoroid neovasculopathy. The refraction was − 3.50 diopters. Best-corrected visual acuity was 0.00 logarithm of the minimum angle of resolution unit. (**A**) Color fundus photograph shows retinal pigment epithelium (RPE) abnormality accompanied by a serous retinal detachment (SRD) at the macular area. (**B**) 12 mm horizontal and vertical B-mode OCT images through the fovea show pachychoroid with dilated outer choroidal vessels (vortex veins). A shallow irregular RPE detachment accompanied by SRD is observed at the fovea. The central choroidal thickness is 401 µm. (**C**,**D**) Fluorescein angiography (early and late phases) shows window defects and some leakage in the macular area. (**E**,**F**) Indocyanine green angiography (early and late phases) shows dilated choroidal vessels and suspected choroidal neovascularization (CNV) at the macular area. Choroidal vascular hyperpermeability is seen around the macular area. (**G**,**H**) OCT angiography (3 mm × 3 mm) shows network vessels comprising CNV between the detached RPE and Bruch’s membrane. En face OCT image (12 mm × 12 mm) shows dilated vortex veins in the deep layer of the choroid. Horizontal watershed is lost due to anastomoses between the superior and inferior vortex veins. (**I**,**J**) En face OCT image (temporal 8 mm × 12 mm), binarized image, and skeletonized image. The area, length, and mean diameter of vortex veins are 57.3  mm^2^, 476 mm, and 120 µm, respectively.

**Figure 4:**
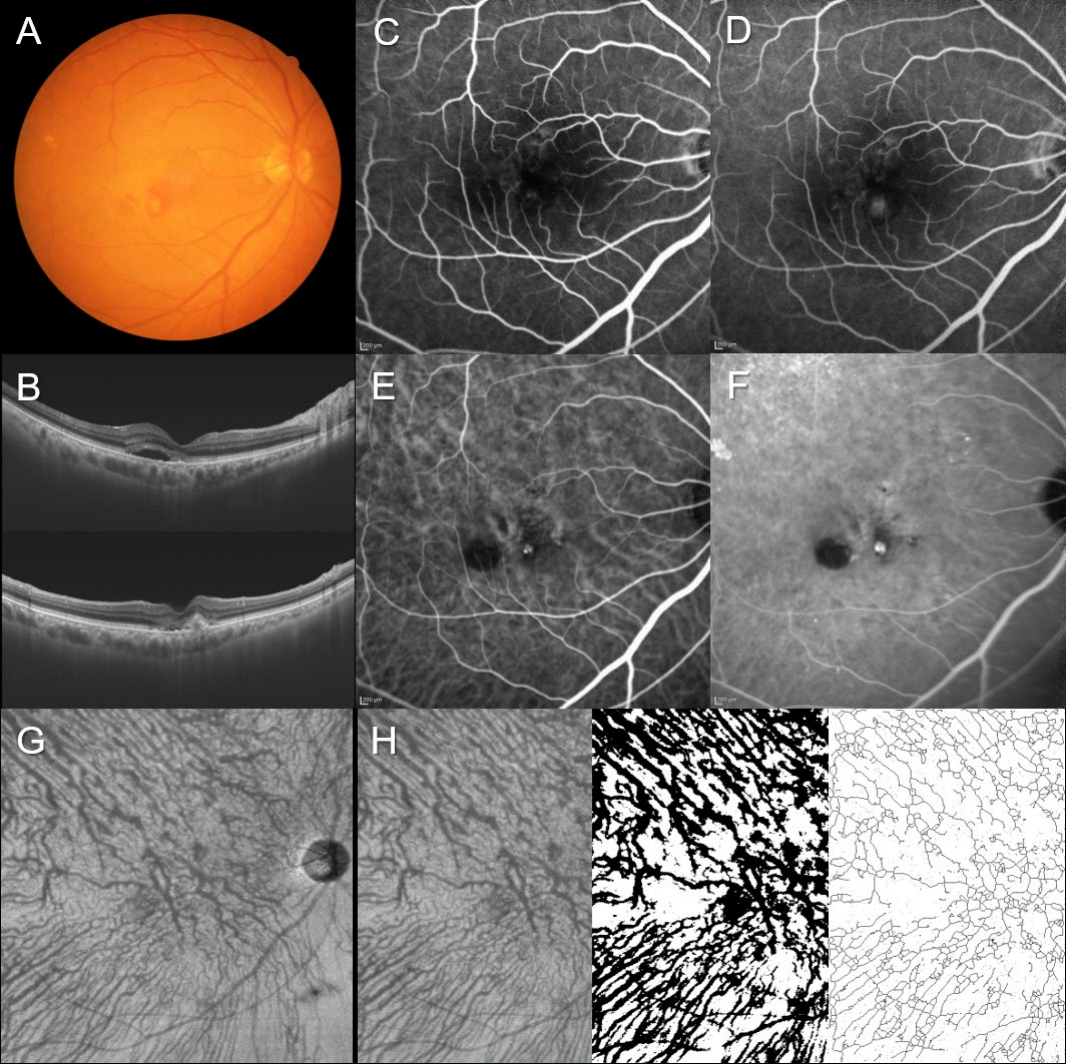
Images of the right eye of an 81-year-old man with polypoidal choroidal vasculopathy. The refraction was − 0.10 diopters. Best-corrected visual acuity was 0.10 logarithm of the minimum angle of resolution unit. (**A**) Color fundus photograph shows retinal pigment epithelium (RPE) detachments accompanied by subretinal hemorrhage and serous retinal detachment (SRD) at the macular area. (**B**) 12 mm horizontal and vertical B-mode optical coherence tomography (OCT) images through the fovea show dilated outer choroidal vessels (vortex veins) associated with RPE detachment and SRD. The central choroidal thickness is 256 µm. (**C**,**D**) Fluorescein angiography (early and late phases) shows window defects in the macular area and some leakage inferior to the fovea. (**E**,**F**) Indocyanine green angiography (early and late phases) shows dilated choroidal vessels and a polypoidal lesion inferior to the fovea. (**G**) En face OCT image (12 mm × 12 mm) shows slightly dilated vortex veins in the deep layer of the choroid. Horizontal watershed is lost because of collateral veins due to anastomoses between the superior and inferior vortex veins. (**H**) En face OCT image (temporal 8 mm × 12 mm), binarized image, and skeletonized image. The area, length, and mean diameter of vortex veins are 47.7  mm^2^, 446 mm, and 107 µm, respectively.

The demographic and clinical characteristics of patients with pachychoroid spectrum diseases are presented in Table 1 and Fig. 1. Cases representative of pachychoroid spectrum diseases are shown in Figs. 2, 3 and 4. The dataset of pachychoroid spectrum diseases analyzed in this study included 94 eyes of 94 patients with CSC, 60 eyes of 60 patients with PNV, and 57 eyes of 56 patients with PCV. The current study included 64 CSC eyes, 41 PNV eyes, and 35 PCV eyes analyzed for our previous publication^16^. The patients with CSC included 79 men (84.0%) and 15 women (16.0%). Their average age was 53.3 ± 14.4 years. Eighty-nine eyes (94.7%) were phakic and 5 eyes (5.3%) had pseudophakia. The refraction of the 89 phakic eyes was − 0.62 ± 1.75 diopters. The patients with PNV included 44 men (73.3%) and 16 women (26.7%). Their average age was 65.2 ± 11.4 years. Fifty-two eyes (86.7%) were phakic and 8 eyes (13.3%) had pseudophakia. The refraction of the 52 phakic eyes was − 0.54 ± 1.81 diopters. The patients with PCV included 45 men (80.4%) and 11 women (19.6%), with an average age of 74.5 ± 8.6 years. Fifty eyes (87.7%) were phakic and 7 eyes (12.3%) had pseudophakia. The refraction of the 50 phakic eyes was 0.02 ± 1.46 diopters. Ages differed significantly among patients with the 3 pachychoroid spectrum diseases (CSC vs PNV, PNV vs PCV, CSC vs PCV: P < 0.01) (Fig. 1A), while there were no significant differences in either gender or refraction.

**Table 1:** Demographic and clinical characteristics of patients with pachychoroid spectrum diseases.

	CSC	PNV	PCV
No. of eyes	94	60	57
Age (years)	53.3 ± 14.4	65.2 ± 11.4	74.5 ± 8.6
Male	79 (84.0%)	44 (73.3%)	45 (80.4%)
Central choroidal thickness (µm)	391 ± 112	315 ± 90	272 ± 108
Vortex vein anastomosis ( +)	87 (92.6%)	57 (95.0%)	56 (98.2%)
Area of vortex veins (mm^2^)	54.8 ± 6.9	52.1 ± 6.7	50.1 ± 5.0
Length of vortex veins (mm)	443 ± 89	436 ± 74	430 ± 74
Mean diameter of vortex veins (µm)	126.6 ± 19.1	121.1 ± 14.3	119.2 ± 18.8

*CSC* central serous chorioretinopathy, *PNV* pachychoroid neovasculopathy without polypoidal lesions, *PCV* polypoidal choroidal vasculopathy (pachychoroid neovasculopathy with polypoidal lesions).

The areas of vortex veins in en face OCT images were 54.8 ± 6.9 mm^2^ in CSC, 52.1 ± 6.7  mm^2^ in PNV, and 50.1 ± 5.0  mm^2^ in PCV. CSC showed a significantly larger vortex vein area than PCV (P < 0.01) (Fig. 1C). The lengths of vortex veins were 443 ± 89 mm in CSC, 436 ± 74 mm in PNV, and 430 ± 74 mm in PCV, showing no significant differences. The mean diameters of vortex veins were 126.6 ± 19.1 µm in CSC, 121.1 ± 14.3 µm in PNV, and 119.2 ± 18.8 µm in PCV. The mean diameter of vortex veins was significantly larger in CSC than in PCV (P < 0.05) (Fig. 1D). CCT was 391 ± 112 µm in CSC, 315 ± 90 µm in PNV, and 272 ± 108 µm in PCV. CCT thus differed significantly among the 3 pachychoroid spectrum diseases (CSC vs PNV: P < 0.01, PNV vs PCV: P < 0.05, CSC vs PCV: P < 0.01) (Fig. 1B). In all 211 eyes, the CCT correlated with the area of vortex veins (rs = 0.44, P < 0.01) and the mean diameter of vortex veins (rs = 0.51, P < 0.01) in en face OCT images. Anastomosis between the superior and inferior vortex veins was observed in 87 eyes (92.6%) with CSC, 57 eyes (95.0%) with PNV, and 56 eyes (98.2%) with PCV. There were no significant differences of the proportion of eyes showing anastomosis among the 3 pachychoroid spectrum diseases.

## Discussion

In our prior study, among patients with pachychoroid spectrum diseases, we found those with CSC to be the youngest, followed by PNV and then PCV patients^16^. The largest CCT values were observed in CSC eyes, followed by PNV eyes and then PCV eyes, while the CCT values from age- and gender-matched controls for each of the pachychoroid spectrum diseases were similar^16^. Moreover, the rate of anastomosis was found to be significantly greater in eyes from subjects with pachychoroid spectrum diseases than those from normal controls^15,16^. The results from the present cohort were consistent with those of our prior study. In the current study, we quantitatively analyzed en face OCT images and measured the areas, lengths, and mean diameters of vortex veins in pachychoroid spectrum diseases. The area and mean diameter of vortex veins were significantly larger in CSC than in PCV, while vortex vein lengths were similar among the three pachychoroid spectrum diseases. Moreover, CCT correlated with the area and mean diameter of vortex veins in all study eyes. These results indicate that while choroidal congestion might be most severe in CSC, this congestion is possibly diminished during the course of progression of pachychoroid spectrum diseases via new drainage routes established by anastomosis between the superior and inferior vortex veins. The area and mean diameter of vortex veins as well as CCT are parameters potentially indicating choroidal congestion.

Warrow et al. first described pachychoroid pigment epitheliopathy as choroidal thickening associated with RPE abnormalities^1^. Pang et al. reported PNV as a form of Type 1 (subretinal pigment epithelium) neovascularization that occurs over areas of increased choroidal thickness and dilated choroidal vessels^5^. They used B-mode OCT images to diagnose pachychoroid spectrum diseases. Dansingani et al. investigated en face OCT images of pachychoroid spectrum diseases and described the dilated outer choroidal vessels as pachyvessels^2^. However, these reports were based on qualitative analysis of OCT images. Moreover, quantitative diagnostic criteria for pachychoroid spectrum diseases have yet to be established. In the current study, we analyzed B-mode and en face OCT images in pachychoroid spectrum diseases quantitatively as well as qualitatively. We demonstrated statistically significant differences not only in CCT but also in the area and mean diameter of vortex veins, i.e. outer choroidal vessels, among the pachychoroid spectrum diseases. Therefore, the binarization method might be useful for analyzing en face OCT images quantitatively. However, it might be difficult to distinguish among the pachychoroid spectrum diseases employing only the parameters obtained from OCT images of the choroid because the values overlapped to some extent among the diseases. Qualitative and quantitative analyses of multimodal imaging will be needed for both accurate diagnosis of and research on pachychoroid spectrum diseases^19^.

Shiihara et al. originally investigated mean vessel diameter in Haller’s layer of the choroid using 7 mm × 7 mm en face OCT images obtained by DRI OCT Triton^10^. The en face images they studied showed mean choroidal vessel diameters of 127 µm in a representative normal eye and 156 µm in a representative CSC eye^10^. While they reported only representative data, the values they obtained are apparently larger than those of our present study. Herein, we used en face OCT images obtained by PLEX Elite 9000, which might account for the difference in the mean choroidal vessel diameter values. Another possible explanation is that we analyzed 12 mm × 12 mm en face OCT images which contained shadow artefacts due to retinal vessels. Shiihara et al. analyzed 7 mm × 7 mm en face OCT images which contained fewer shadow artefacts due to the retinal vessels than 12 mm × 12 mm en face OCT images. The mean diameter of vortex veins in the current study might have been lower than the true values because retinal vessels are usually thinner than choroidal vessels. However, the area and length of retinal vessels were similar in en face OCT images of all of the eyes in this study. Therefore, comparisons of choroidal vessel diameters among the pachychoroid spectrum diseases might be limitedly affected by shadow artefacts due to retinal vessels.

The limitations of this study include that it was retrospective and performed at a single center. The axial length was not quantified in all cases. OCT was performed by focusing on the posterior pole of the fundus, and as such, only the posterior portion of the choroidal circulation was assessed. Such a limited area cannot explain the entire vortex vein system because only the terminal tributaries of the vortex veins were analyzed. En face OCT images for the analysis were selected subjectively at the depth where the vortex veins showed maximal dilatation. The presence of anastomosis between the superior and inferior vortex veins on en face OCT images was also judged in a subjective manner. All subjects were Japanese, and therefore the results may not be generalizable to pachychoroid spectrum diseases in other ethnic groups.

In conclusion, the binarization method was useful for quantitatively evaluating en face OCT images in eyes with pachychoroid spectrum diseases. New drainage routes formed by anastomosis developing between the superior and inferior vortex veins might compensate for the choroidal congestion characteristic of pachychoroid spectrum diseases.

## Methods

We performed this study, which complied with the guidelines of the Declaration of Helsinki, with approval from Institutional Review Board of Gunma University Hospital. Informed consent was obtained from all individual participants included in the study. We retrospectively studied 347 eyes of 335 patients with treatment naïve pachychoroid spectrum diseases including CSC and PNV with or without polypoidal lesions, followed clinically from April 2017 through March 2020 at Gunma University Hospital. We excluded 136 eyes of 125 patients with low quality en face OCT images from the analysis. All patients with pachychoroid spectrum diseases underwent a complete ophthalmological examination, including color fundus photography (Canon CX-1; Canon, Tokyo, Japan), fluorescein angiography (FA) and ICGA with an angle of 30 degrees (Spectralis HRA + OCT; Heidelberg Engineering, Heidelberg, Germany), as well as swept-source OCT (DRI OCT-1 Triton; Topcon Corp, Tokyo, Japan, and PLEX Elite 9000; Carl Zeiss Meditec, Dublin, CA, USA)^16^. We obtained B-mode images of the horizontal and vertical line scans (12 mm) through the fovea employing the DRI OCT-1 Triton^16^. Next, cube data were obtained with a raster scan protocol of 1024 (horizontal) × 1024 (vertical) B-scans, which covered the 12 × 12 mm area centered on the fovea by the PLEX Elite 9000^16^. En face images were obtained from the vitreous to the choroidoscleral border with coronal slices from a 3-dimensional dataset included in the inner software. Then, we performed OCT angiography (OCTA) volume scanning, i.e. 300 × 300 pixels in the 3 × 3 mm area demonstrated by the PLEX Elite 9000^16^. The OCTA thus performed was based on an optical microangiography algorithm.

Herein, clinical and anatomical features of the pachychoroid were defined as pathologically dilated outer choroidal vessels on B-mode or en face OCT images. We diagnosed CSC if all of the following criteria were met. (1) Pachychoroid was accompanied by serous retinal detachment (SRD). (2) FA showed dye leakage within the SRD. (3) CNV was ruled out by FA, ICGA, and OCTA images. Pachychoroid neovasculopathy was diagnosed if CNV associated with pachychoroid was detected by FA, ICGA, and/or OCTA images. CNV findings on OCTA were detected in the slab from the outer retina to the choriocapillaris. The presence of polypoidal lesions was evaluated on ICGA and B-mode OCT images, i.e. polyp-like choroidal vessel dilation on ICGA and sharply peaked retinal pigment epithelium (RPE) detachment on B-mode OCT^20^. In this study, PNV meant PNV without polypoidal lesions, while PCV meant PNV with polypoidal lesions.

We first evaluated the CCT on B-mode OCT images and the presence of anastomosis between the superior and inferior vortex veins using en face OCT images in eyes with pachychoroid spectrum diseases^15,16^. En face OCT images obtained at successive depths of 8 µm in the choroid were assessed. Vortex vein anastomosis was considered to be present if anastomotic vessels connected the superior and inferior vortex veins. The anastomotic vessels did not show narrowing toward the watershed zone. The CCT and the presence of anastomosis between the superior and inferior vortex veins on en face OCT images were judged by two experienced retinal specialists (H. Matsumoto and J. Hoshino), working independently of each other. Next, we selected an en face OCT image at the depth where the vortex veins were most dilated from each eye. A temporal 8 mm × 12 mm area of the image was used for assessing the area and length of vortex veins because the nasal 4 mm × 12 mm area included the optic nerve and thin choroid around the optic nerve. The area and length of vortex veins were calculated according to a previous report^10^. Briefly, 8 mm × 12 mm en face OCT images were processed by a software program with the C++ programming language. The histogram was flattened by contrast-limited adaptive histogram equalization. The image noise was removed by a non-local mean filter. Then, each image was binarized to calculate the area of vortex veins. Next, a thinning of the vessel was performed by applying the Zhang-Suen thinning algorithm to calculate the length of vortex veins as the total length of the line. Finally, we determined the mean diameter of vortex veins in eyes with pachychoroid spectrum diseases by dividing the area of vortex veins by their length.

For statistical analyses, the Mann–Whitney U test was used to compare unpaired values of age. The chi-squared independence test was used to determine differences in gender and the incidence of anastomosis between the superior and inferior vortex veins. The Tukey–Kramer test or Steel–Dwass test was used for multiple comparison analyses of age, refraction, CCT, and the area, length, and mean diameter of vortex veins. The Spearman rank correlation coefficient was calculated to study the associations between CCT and the area, length, and mean diameter of vortex veins. These data analyses were performed using Excel 2016 (Microsoft, Redmond, WA, USA) with add-in software Statcel4 (OMS, Tokyo, Japan)^21^. A P < 0.05 was considered to indicate a statistically significant difference. All data are presented as the average ± standard deviation.
